# Genomic study of *Acinetobacter baumannii* strains co-harboring *bla*_OXA-58_ and *bla*_NDM-1_ reveals a large multidrug-resistant plasmid encoding these carbapenemases in Brazil

**DOI:** 10.3389/fmicb.2024.1439373

**Published:** 2024-07-17

**Authors:** Daiana Cristina Silva Rodrigues, Melise Chaves Silveira, Bruno Rocha Pribul, Bruna Ribeiro Sued Karam, Renata Cristina Picão, Gabriela Bergiante Kraychete, Felicidade Mota Pereira, Rildo Mendes de Lima, Antonio Kleber Gomes de Souza, Robson Souza Leão, Elizabeth Andrade Marques, Cláudio Marcos Rocha-de-Souza, Ana Paula D'Alincourt Carvalho-Assef

**Affiliations:** ^1^Laboratório de Bacteriologia Aplicada à Saúde Única e Resistência Antimicrobiana (LabSUR), Instituto Oswaldo Cruz (IOC), Fundação Oswaldo Cruz (Fiocruz), Rio de Janeiro, Brazil; ^2^Laboratório de Investigação em Microbiologia Médica (LIMM), Instituto de Microbiologia Paulo de Góes (IMPG), Universidade Federal do Rio de Janeiro (UFRJ), Rio de Janeiro, Brazil; ^3^Laboratório Central de Saúde Pública do Estado da Bahia (LACEN-BA), Bahia, Brazil; ^4^Laboratório Central de Saúde Pública da Fundação de Vigilância em Saúde do Amazonas (LACEN-AM/FVS-RCP), Amazonas, Brazil; ^5^Laboratório Reunidos Ltda., Amazonas, Brazil; ^6^Departamento de Microbiologia, Imunologia e Parasitologia (DMIP), Faculdade de Ciências Médicas (FCM), Universidade do Estado do Rio de Janeiro (UERJ), Rio de Janeiro, Brazil

**Keywords:** *Acinetobacter baumannii*, OXA-58 carbapenemase, NDM-1 carbapenemase, genomic, plasmids, co-harboring

## Abstract

**Introduction:**

*Acinetobacter baumannii* contributes significantly to the global issue of multidrug-resistant (MDR) nosocomial infections. Often, these strains demonstrate resistance to carbapenems (MDR-CRAB), the first-line treatment for infections instigated by MDR *A. baumannii*. Our study focused on the antimicrobial susceptibility and genomic sequences related to plasmids from 12 clinical isolates of *A. baumannii* that carry both the blaOXA-58 and *bla*_NDM-1_ carbapenemase genes.

**Methods:**

Whole-genome sequencing with long-read technology was employed for the characterization of an A. baumannii plasmid that harbors the *bla*_OXA-58_ and blaNDM-1 genes. The location of the *bla*_OXA-58_ and *bla*_NDM-1_ genes was confirmed through Southern blot hybridization assays. Antimicrobial susceptibility tests were conducted, and molecular characterization was performed using PCR and PFGE.

**Results:**

Multilocus Sequence Typing analysis revealed considerable genetic diversity among *bla*_OXA-58_ and *bla*_NDM-1_ positive strains in Brazil. It was confirmed that these genes were located on a plasmid larger than 300 kb in isolates from the same hospital, which also carry other antimicrobial resistance genes. Different genetic contexts were observed for the co-occurrence of these carbapenemase-encoding genes in Brazilian strains.

**Discussion:**

The propagation of *bla*_OXA-58_ and *bla*_NDM-1_ genes on the same plasmid, which also carries other resistance determinants, could potentially lead to the emergence of bacterial strains resistant to multiple classes of antimicrobials. Therefore, the characterization of these strains is of paramount importance for monitoring resistance evolution, curbing their rapid global dissemination, averting outbreaks, and optimizing therapy.

## 1 Introduction

*Acinetobacter baumannii* has emerged as one of the most significant and challenging pathogens of this century, primarily due to its involvement in multidrug-resistant (MDR) hospital-acquired infections (Zhang et al., 2021). The most common clinical presentations linked to this pathogen include ventilator-associated pneumonia (VAP) and bloodstream infections (Palmieri et al., 2020). The management of *Acinetobacter* infections is notably difficult due to its exceptional capacity to develop resistance to nearly all classes of antimicrobials currently used in clinical settings, particularly carbapenems (Ghaly et al., 2020), which are the treatment of choice for MDR *A. baumannii* (MDR-Ab) infections. However, the distressing increase in carbapenem-resistant *A. baumannii* (CRAB) strains globally has significantly limited treatment alternatives and considerably increased morbidity and mortality rates (Kyriakidis et al., 2021). Importantly, CRAB strains often demonstrate co-resistance to other antibiotic classes, further complicating therapeutic approaches (Nodari et al., 2020).

CRAB is primarily linked with the production of carbapenem-hydrolyzing class D β-lactamases (CHDL), also referred to as oxacillinases (OXA). At present, among the recognized phylogenetic subgroups of CHDL, five are commonly identified in *A. baumannii*: intrinsic chromosomal OXA-51-like, acquired OXA-23-like, OXA-24-like, OXA-58-like, and OXA-143-like (Goic-Barisic et al., 2021). These carbapenemases are generally expressed at low levels; however, the presence of an insertion sequence (IS) element upstream of these genes, such as IS*Aba1*, serves as a powerful transcriptional promoter, leading to clinical carbapenem resistance (Wong et al., 2017). Among these enzymes, OXA-23-like is the most widespread and frequently reported enzyme worldwide (Goic-Barisic et al., 2021). In Brazil, OXA-58 is detected sporadically (Matos et al., 2019), while OXA-23 is prevalent, followed by OXA-143 (Vasconcelos et al., 2015; de Oliveira et al., 2019). The OXA-24-like and OXA-58-like enzymes appear to be endemic in specific regions of the world (Hamidian and Nigro, 2019).

Carbapenem resistance in *Acinetobacter* spp. can also occur. However, it is less common due to the production of metallo-β-lactamases (MBL), such as imipenemase (IMP), Verona integron-encoded metallo-β-lactamase (VIM), and New Delhi metallo-β-lactamase (NDM) (Anggraini et al., 2022). Notably, NDM-1 is prevalent in *Klebsiella pneumoniae* and *Escherichia coli* species, whereas *Acinetobacter* spp. is known as an intermediate reservoir. However, NDM-1-producing *Acinetobacter* strains have been reported globally due to the high horizontal transferability of the plasmids carrying the *bla*_NDM_ gene and several additional resistance mechanisms, restricting treatment options (Vasudevan et al., 2022).

The co-occurrence of two distinct carbapenemase-encoding genes is a particularly alarming mechanism of antimicrobial resistance, as it typically results in higher resistance to β-lactams and is often associated with increased mortality rates (Oinuma et al., 2016; Vasudevan et al., 2022).

In this study, we examined antimicrobial susceptibility and investigated the genomic sequences associated with plasmids from *A. baumannii* clinical isolates carrying the *bla*_OXA-58_ and *bla*_NDM-1_ genes, with the aim of gaining a deeper understanding of the molecular basis and evolutionary dynamics of the antimicrobial resistance of isolates circulating in Brazil.

## 2 Materials and methods

### 2.1 Bacterial isolates

The Laboratório de Bacteriologia Aplicada à Saúde Única e Resistência Antimicrobiana (LabSUR-Fiocruz) receives clinical isolates from public health laboratories across Brazil to investigate the mechanisms of antimicrobial resistance. The present study encompasses 12 unique clinical isolates of OXA-58- and NDM-1-producing *A. baumannii*, which are part of the Culture Collection of Hospital-Acquired Bacteria (CCBH-Fiocruz, WDCM 947). These isolates were obtained from various clinical specimens, including blood, tracheal secretions, catheter tips, and cerebrospinal fluid, from patients hospitalized in two Brazilian states: Bahia in the northeast (eight isolates from two hospitals) and Amazonas in the north (four isolates from three hospitals). The strains were isolated over a period of 1 year, from November 2020 to November 2021, and were sent to LabSUR for investigation into the molecular mechanisms of carbapenem resistance. During this same period, the laboratory received 1,124 strains of *Acinetobacter* spp. After performing a polymerase chain reaction (PCR), the *bla*_OXA-58_ and *bla*_NDM-1_ genes were detected in 12 isolates.

### 2.2 Antimicrobial susceptibility testing

The antimicrobial susceptibility profile was performed and interpreted according to the European Committee on Antimicrobial Susceptibility Testing (EUCAST) guidelines (European Committee on Antimicrobial Susceptibility Testing (EUCAST), 2023). Antimicrobial susceptibility testing (AST) of *A. baumannii* isolates for amikacin, gentamicin, ciprofloxacin, trimethoprim–sulfamethoxazole, tobramycin, levofloxacin, and meropenem was performed using the Kirby–Bauer disk-diffusion method, as described previously (Bauer et al., 1966). The broth dilution method was used to determine the susceptibility to colistin.

### 2.3 Pulsed-field gel electrophoresis

All the isolates were analyzed using PFGE, as described previously, with minor modifications (Bou et al., 2000). Bacterial cells were embedded in agarose plugs and digested by *ApaI* (Invitrogen) at 37°C for 3 h. Electrophoresis was performed on 1.1% agarose gel (SeaKem^®^ Gold Agarose, Lonza) in 0.4× Tris-borate-EDTA buffer using a CHEF-DR III System (Bio-Rad) apparatus. Images of banding patterns obtained were processed using BioNumerics software (version 6.6; Applied Maths). Similarities were calculated using both Dice coefficients, and the unweighted pair-group method using arithmetic averages (UPGMA) was applied for cluster analysis. The tolerance and optimization were set at 1.5% each. Strains were considered epidemiologically related if they had ≥90% genetic similarity.

Plasmids were analyzed using S1 nuclease (Invitrogen) digestion followed by Southern blot hybridization for all 12 strains. After transfer to Amersham Hybond-N+ membranes (GE Healthcare), the genomic DNA was hybridized with the *bla*_OXA-58_ and *bla*_NDM-1_ probes as described in the DIG-DNA labeling and detection kit (Roche Diagnostics, Germany).

### 2.4 Whole-genome sequencing

Only CCBH31258 was subjected to long-read sequencing by Oxford Nanopore Technologies^®^ (ONT). Genomic DNA was extracted using the DNeasy^®^ PowerSoil^®^ Pro Kit (Qiagen), following the manufacturer’s recommendations. The barcoding sequencing library was prepared according to the protocol for native barcoding genomic DNA using the Ligation Sequencing SQK-LSK109 and Native Barcoding Expansion 1–12 EXP-NBD104 kits (Oxford Nanopore Technologies^®^), as well as the NEB Blunt/TA Ligase Master Mix, NEBNext^®^ Quick Ligation Reaction Buffer, and NEBNext^®^ Companion Module for Oxford Nanopore Technologies^®^ Ligation Sequencing. After that, the DNA library was loaded onto a FLO-MIN106D flow cell (R9.4 chemistry) to run on Oxford Nanopore MinION. MinKNOW v.21.02.1 was used to obtain sequence signals, while basecalling and demultiplexing were performed using Guppy basecaller v1.6.0.

Based on the PFGE analysis, all isolates except CCCBH31258 were subjected to Illumina whole-genome sequencing. Genomic DNA from each strain from the overnight culture was extracted using the QIAamp DNA mini kit (Qiagen), following the manufacturer’s instructions. A tagmentation library from genomic DNA was made using the Nextera XT DNA Sample Preparation Kit (Illumina, San Diego, CA, United States), and the 250-bp paired-end reads were sequenced on the Miseq system (Illumina, San Diego, CA, United States).

#### 2.4.1 Assembly

The *de novo* assembly for CCBH31258 long reads was generated using the Flye assembler (Kolmogorov et al., 2019). For the short reads from the other seven strains, Trimmomatic was used at the trimming step (ILLUMINACLIP:NexteraPE-PE.fa:2:30:10 AVGQUAL:20 MINLEN:50) (Bolger et al., 2014). The genome *de novo* assembly was performed using Unicycler (v0.4.9), conservative mode (Wick et al., 2017). Short reads were mapped against plasmids from CCBH31258 (pCCBH31258) using minimap2 and SAMtools (Li et al., 2009; Li, 2018). For strains that read coverage for all pCCBH31258, a plasmid consensus sequence was generated using Pilon, and VCF output was checked (Walker et al., 2014). Furthermore, contigs were mapped against the plasmid consensus using minimap2, and the mapped contigs were excluded to obtain only chromosome contigs. Contigs from the other strains were extended by AlignGraph (Bao et al., 2014), using pCCBH31258 as a reference. For each strain, mapped reads (SAM files) using pCCBH31258 as a reference were compared.

#### 2.4.2 Annotation

Draft genomes were submitted to GenBank and annotated using the NCBI Prokaryotic Genome Annotation Pipeline (PGAP). The web application CABGen was used to access coverage estimation; species confirmation; MLST mapping; searches for genes related to AMR, virulence, and plasmids; and detection of point mutations in specific AMR genes (Duré et al., 2022). Resistance genes were confirmed using raw reads as input for ResFinder software on the Center for Genomic Epidemiology website. Insertion sequence annotation was improved in the figures using ISFinder (Siguier, 2006). MOB-suite was applied to the assembled plasmid to predict the Inc. group, relaxase typing, and conjugation potential (Robertson and Nash, 2018). PdifFinder analyses were adopted for the annotation of *pdif* sites and *pdif*-ARGs modules in the plasmid (Shao et al., 2023).

## 3 Results

### 3.1 Antimicrobial susceptibility profile

**Figure 1:**
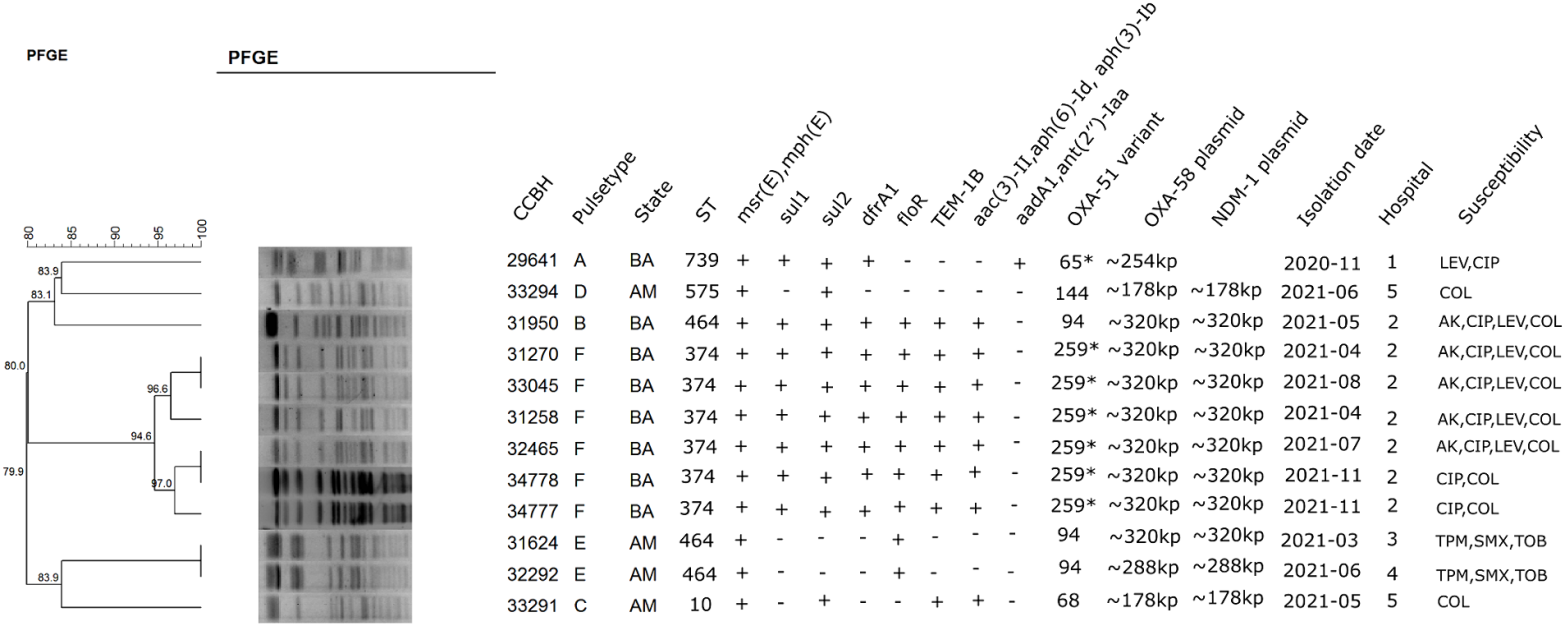
Dendrogram cluster analysis of PFGE data with a ≥ 90% cut-off level of 12 clinical isolates of *Acinetobacter baumannii* and other relevant information about the strains. BA: Bahia; AM: Amazonas; AK: amikacin; CIP: ciprofloxacin; TMP/SMX: trimethoprim–sulfamethoxazole; TOB: tobramycin; LEV: levofloxacin; COL: colistin. *99% identity.

The isolates exhibited a resistance profile to the majority of the antimicrobials tested, with the highest resistance rates to meropenem (100%), gentamicin (100%), trimethoprim–sulfamethoxazole (83%), and tobramycin (83%). Colistin was the most active agent tested, with only two resistant isolates, one from Bahia and the other from Amazonas (Figure 1).

### 3.2 Pulsed-field gel electrophoresis

The 12 strains were grouped into six PFGE clusters (A, B, C, D, E, and F), and the most frequent pulsetype was pattern F (six strains from the same hospital in Bahia), followed by pattern E (2 strains from two different hospitals in Amazonas). In Bahia and Amazonas, two other pulsetypes were found (A and B in Bahia and C and D in Amazonas). Southern blot hybridization showed that *bla*_OXA-58_ and *bla*_NDM-1_ genes were located on a plasmid with approximately 320 kb for clone B and clone F strains, which were isolated in the Bahia state, in the same hospital. Furthermore, two isolates from the Amazonas state, belonging to clone E, showed labeled bands with similar weights (CCBH31624 and CCBH32292). Two strains from Amazonas (CCBH33291 and CCBH33294), isolated in the same hospital, showed the same labeled band of approximately 178 kb, although they belong to different clones (C and D) (Figure 1; Supplementary material).

### 3.3 Whole-genome sequencing

The *de novo* assembly for CCBH31258 (pulsotype F) long reads generated two contigs: one chromosome with 3,706,655 bp and one circularized plasmid with 340,418 bp (GenBank accession CP101889-CP101888). This isolate belongs to ST374 and carries *bla*_OXA-259_ (a *bla*_OXA-51_ variant). Both carbapenemase-encoding genes (*bla*_OXA-58_ and *bla*_NDM-1_) were detected in the MDR plasmid (pCCBH31258), which also carried resistance genes to several other antimicrobials, such as *bla*_TEM-1B_, *aac(3)-IIa*, *aph(6)-Id*, *msr(E)*, *mph(E)*, *dfrA1*, *floR*, *sul1*, and *sul2* (Figure 1).

**Figure 2:**
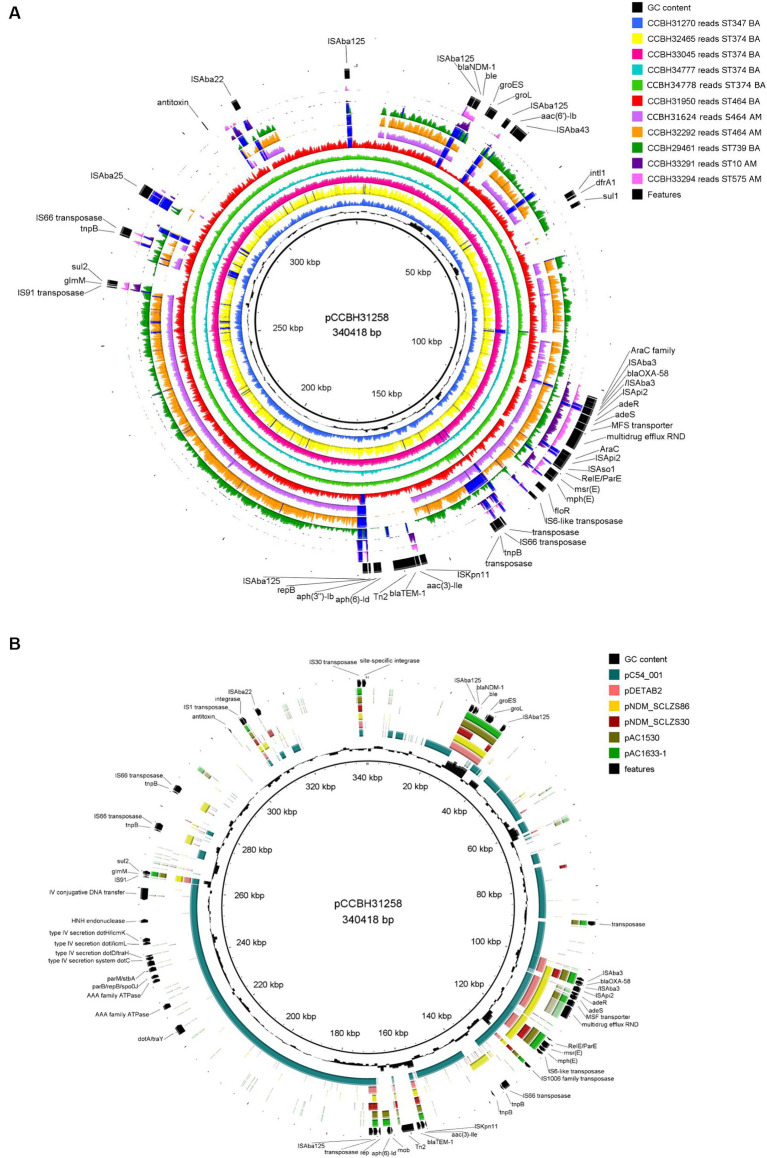
Genomic comparison of plasmid pCCBH31258 with **(A)** other *Acinetobacter baumannii* strains from this study and **(B)** selected plasmids from GenBank: pC54_001 (CP042365.1), pDETAB2 (CP047975.1) (Liu et al., 2021), pNDM_SCLZS86 (CP090865.1) (Li et al., 2022b), pNDM_SCLZS30 (CP090384.1) (Li et al., 2022a), pAC1530 (CP045561.1) (Alattraqchi et al., 2021), and pAC1633-1 (CP059301) (Alattraqchi et al., 2021). Inside out, the first ring is the plasmid pCCBH31258, used as a reference (CP101888.1). In **(A)**, each strain was represented by its reads mapping coverage against the reference plasmid. The “graph maximum value” was set to 400x, which represents the maximum coverage peak shown. Regions with coverage over 400x were highlighted in dark blue. In **(A,B)**, the last ring represents relevant features in the reference. BA: Bahia state; AM: Amazonas state.

In plasmid pCCBH31258, the *bla*_NDM-1_ gene was identified in a DNA region characterized by a distinct guanine-cytosine (GC) content compared to the reads of the other isolates analyzed. This region also encompasses genes encoding a chaperone and a bleomycin-binding protein, corresponding to the complete Tn*125* transposon, flanked by IS*Aba125* (10,099 bp) (Figure 2A).

The *bla*_OXA-58_ was inserted between a complete IS*Aba3* upstream and a truncated IS*Aba3* downstream, followed by a complete IS*Api2*. This structure belongs to a *pdif*-ARGs module in pCCBH31258, surrounded by inversely oriented p*dif* sites (XerC/D and XerD/C). Surrounding *bla*_OXA-58_, *araC* family gene and genes related to efflux systems (*adeR*, *adeS*, MFS transporter, and multidrug efflux RND) were identified. Macrolide resistance genes, *msr(E)* and *mph(E),* were also located next to *bla*_OXA-58_ (Figure 2A).

Two genomic comparisons were applied: mapped reads from this study strains (Figure 2A) and GenBank assembly plasmids co-harboring *bla*_OXA-58_ and *bla*_NDM-1_, both using pCCBH31258 as a reference (Figure 2B). After visual inspection of the mapping results, reads from CCBH31270, CCBH32465, CCBH33045, CCBH33777, and CCBH33778 (typed as pulsotype F and ST374) covered all regions of pCCBH31258 (Figure 2A). Therefore, for these strains, the plasmid consensus assembly was adopted, and it was assumed that they carried the same plasmid (CP101886.1, JAPDGP000000000.1, JBDJPK000000000.1, JBDJPJ000000000.1, and JBDJPI000000000.1). Reads from CCBH31950 (pulsotype B, ST464) covered almost all pCCBH31258, missing only specific IS regions (IS*Api2* and IS*Aba22*) (Figure 2A). These seven strains were isolated in the same hospital in Bahia state, Brazil, between April and July 2021.

Although the weight estimation derived from hybridization results suggested that CCBH31624, isolated in Amazonas, harbored a plasmid similar to pCCBH31258, sequencing results indicated distinct gene sets (Figure 1). According to the mapping results and gene content, CCBH31624 and CCBH32292 (both pulsotype E, ST464) appear to carry a plasmid with a genomic background different from pCCBH31258, despite the similar weight observed in the Southern blot. For CCBH29641 (pulsotype A, ST739), retrieved from a different hospital in Bahia, the mapping results also demonstrated a genomic background distinct from pCCBH31258. Two sequenced strains from the same hospital in Amazonas (CCBH33291 and CCBH33294), belonging to ST10 and ST575, respectively, exhibited minimal similarities with pCCBH31258. For all these five strains, Tn*125* harboring *bla*_NDM-1_ was incomplete (Figure 2A).

Consequently, for STs other than ST374, it could not be confirmed by sequencing that *bla*_OXA-58_ and *bla*_NDM-1_ were inserted in the same plasmid, despite indications from Southern blot results. Given this, the contigs output from Align-Graph was adopted. For all isolates in this study, only the upstream insertion ISAba125 and downstream bleomycin gene were conserved surrounding *bla*_NDM-1_, and only the IS*Aba3* upstream and downstream regions were conserved for *bla*_OXA-58_ (Figure 2A).

MobTyper analysis revealed that pCCBH31258 encodes a relaxase that belongs to the MOBp family and was thus classified as a mobilizable plasmid. The Inc. group could not be determined; only a *repB* gene was identified (KT325596, 23,590–24,066), which was not included in the rep families characterized by Lam and Hamidian (2024). Furthermore, MobTyper identified pC54_001 (CP042365, *A. pittii*, clinical origin, Australia) as the closest plasmid in its database, sharing 99% identity (65% coverage). A literature search revealed other plasmids co-harboring OXA-58 and NDM-1, including pDETAB2 (CP047975.1), pNDM_SCLZS86 (CP090865.1), pNDM_SCLZS30 (CP090384.1), pAC1530 (CP045561.1), and pAC1633-1 (CP059301). A comparison of pCCBH31258 with these plasmids revealed that the former is a novel plasmid carrying these carbapenemase genes together. pCCBH31258 likely evolved from a *bla*_OXA-58_ plasmid, as it shares conjugation genes from the type IVB secretion system and other backbone genes (*parB* and *parM*) with pC54_001. Moreover, the replicon gene (*rep*) of pCCBH31258 encoded a replicase protein different from those present in the *A. baumannii* plasmids used for comparison (Figure 2B). Two strains from Amazonas, CCBH33291 and CCBH33294, which exhibited a similarly labeled band with an approximate size of 178 kb, were analyzed by mapping their reads against pAC1530 and pAC1633-1. These plasmids share a similar size, but the read coverage was found to be below 20%.

## 4 Discussion

*Acinetobacter baumannii* exhibits an extraordinary ability to develop resistance to multiple antimicrobial agents, particularly through mobile genetic elements, such as plasmids, that harbor antimicrobial resistance determinants. The prevalence of multidrug-resistant *A. baumannii* (MDR-Ab) infections in hospital settings is a significant health concern, especially regarding last-resort antibiotics such as carbapenems. In the present study, all *bla*_OXA-58_ and *bla*_NDM-1_ positive strains were resistant to meropenem and exhibited the MDR phenotype, aligning with the global increase in MDR-Ab infections (Nguyen and Joshi, 2021).

Carbapenem-resistant *A. baumannii* (CRAB) is a therapeutic challenge because few currently available antibiotics are active against CRAB (Bartal et al., 2022). Carbapenem resistance makes colistin the last-resort antimicrobial to treat MDR-Ab infections. However, overuse of this antimicrobial to treat CRAB infections may have contributed to the increased prevalence of colistin-resistant *A. baumannii* (Kurihara et al., 2022). Although most of the strains in the current study were susceptible to colistin, resistance was observed in two isolates.

CRAB is generally associated with a wide range of co-resistance to other classes of antimicrobial agents. The production of OXA-type carbapenemases is the main mechanism of carbapenem resistance in *A. baumannii*. These carbapenemases are encoded by the *bla*_OXA_ genes, which are usually carried by highly transmissible plasmids (Nguyen and Joshi, 2021). In Brazil, *bla*_OXA-23_ is the most widely disseminated OXA-type carbapenemase in CRAB, followed by *bla*_OXA-143_, while *bla*_OXA-58_ is less common (de Souza Gusatti et al., 2012; de Oliveira et al., 2019; Matos et al., 2019).

OXA-58 was first described in 2003 in France; since then, it has been widely reported worldwide, being associated with outbreaks of nosocomial infections (Moro et al., 2008; Ozen et al., 2009; de Souza Gusatti et al., 2012). OXA-58 hydrolyzes carbapenems at low levels; however, expression can be increased by the presence of insertion sequences, such as IS*Aba3*, resulting in resistance to carbapenems (Walther-Rasmussen and Høiby, 2006; Evans and Amyes, 2014). In the present study, all representative isolates sequenced showed complete IS*Aba3* upstream of the *bla*_OXA-58_ gene, suggesting its higher expression. The *bla*_OXA-58_ gene was inserted in a *pdif* module flanked by inversely oriented p*dif* sites in pCCBH31258, suggesting that this gene was probably mobilized and mediated by p*dif* sites using a XerC-XerD recombination system (Li et al., 2022a).

The emergence of NDM in CRAB has also become an important public health issue, which remains a major challenge for the treatment of infectious diseases. NDM is reported in most regions of the world due to its rapid dissemination since the gene encoding NDM is often carried by transferable plasmids (Villacís et al., 2019). pCCBH31258 analyzed in this study revealed that the *bla*_NDM-1_ gene was located between two copies of the IS*Aba125* element, and all strains carried the *ble*_MBL_ (bleomycin resistance) gene, which has always been identified downstream of the *bla*_NDM-1_ gene. Several studies reported that the genetic environment of the *bla*_NDM-1_ gene presents a conserved structure, where this gene is located between two copies of the IS*Aba125* element, forming a transposon named Tn*125* (Dortet et al., 2014; Pagano et al., 2016). However, it could be observed in this study that the Tn*125* complete structure was conserved only for the strains that harbored the complete plasmid characterized here.

Our research describes 12 strains of carbapenem-resistant *Acinetobacter baumannii* (CRAB) concurrently carrying *bla*_OXA-58_ and *bla*_NDM-1_, representing 1% of all *Acinetobacter* species received by the laboratory during the period. The coexistence of these carbapenemase genes in CRAB has been infrequently reported. There have been four instances of CRAB isolates co-producing OXA-58 and NDM-1 from Algeria (Ramoul et al., 2016), Japan (Oinuma et al., 2016), Malaysia (Alattraqchi et al., 2021), and China (Liu et al., 2021). Only the strains isolated in Malaysia and China have the *bla*_OXA-58_ and *bla*_NDM-1_ genes located on the same plasmid (Alattraqchi et al., 2021; Liu et al., 2021) (Figure 2B), while in the Algerian isolate, the genes were chromosomally located (Ramoul et al., 2016), and the genome of the Japanese strain is a draft (Oinuma et al., 2016). Other non-*baumannii Acinetobacter* carrying *bla*_OXA-58_ and *bla*_NDM-1_ genes have been reported from Malaysia (Alattraqchi et al., 2021), China (Zhou et al., 2015; Chen et al., 2019; Li et al., 2022a,b), and Palestine (Regeen et al., 2014). Of these reports, three isolates were sequenced and found to carry these genes on the same plasmids (Alattraqchi et al., 2021; Li et al., 2022a,b) (Figure 2B). To the best of our knowledge, this is the first study reporting *A. baumannii* strains in Brazil co-harboring *bla*_OXA-58_ and *bla*_NDM-1_ genes. Genomic analysis revealed that pCCBH31258 harbored genes coding for proteins with high-level similarity to components of the Dot/Icm type IV secretion system from species of *Legionella* and *Coxiella* (type IVB) (Segal et al., 2005).

It could be noted that there is a high level of ST diversity associated with the co-occurrence of *bla*_OXA-58_ and *bla*_NDM-1_ in Brazil, which suggests that this co-occurrence does not relate exclusively to the clone. MLST analysis showed that five different sporadic STs were present in 12 whole-genome sequenced strains: ST10, ST374, ST464, ST739, and ST575. Interestingly, none of the STs described here belonged to the main clonal complexes CC1, CC15, CC25, and CC79 predominant in South America or to CC2, which is the most prevalent clonal lineage in the world (Rodríguez et al., 2018). The same diversity of plasmids co-harboring *bla*_OXA-58_ and *bla*_NDM-1_ can also be highlighted, considering isolates from other countries (Alattraqchi et al., 2021; Liu et al., 2021; Li et al., 2022a,b).

The most frequent ST was ST374, including six isolates from the same hospital, and all these strains carried the same plasmid cohabiting *bla*_OXA-58_/*bla*_NDM-1_. The *A. baumannii* ST374 has rarely been reported (Fedrigo et al., 2022), and only one study from Tanzania reported a meropenem-resistant isolate, belonging to ST374, which was found to harbor *bla*_NDM-1_ in a chromosomally located composite transposon *Tn*125 (Moyo et al., 2021), while our observations revealed a plasmid localization for the *bla*_NDM-1_ gene.

A very similar plasmid to the one characterized here was observed in an isolate in the same hospital, belonging to ST464 (CCBH31950). Hybridization approaches suggest that another plasmid (~178 kb) is responsible for the mobilization of the two carbapenemase-encoding genes in strains from different clones but isolated in the same hospital in Amazonas state. Horizontal transfer enables different clonal lineages to acquire the same accessory genetic elements (Valenzuela et al., 2007). Furthermore, the mobilization of both genes on the same plasmid may develop an increasing number of OXA-58/NDM-1 co-producer isolates. This scenario is favored by the high plasticity that the genome of *A. baumannii* has in acquiring, retaining, and disseminating antimicrobial resistance genes, especially in plasmids, which play a key role in harboring and transferring antibiotic-resistant genes (Roca et al., 2012).

## 5 Conclusion

Our study delineates a large plasmid harboring OXA-58 and NDM-1 carbapenemases, which have disseminated among clonal strains isolated during 2021 in the same hospital. These strains have accumulated other antimicrobial resistance genes. Furthermore, other genetic contexts have also demonstrated the capacity to disseminate *bla*_OXA-58_ and *bla*_NDM-1_. Our limited access to ONT sequencing prevented the complete genomic characterization of all plasmids harboring *bla*_OXA-58_ and *bla*_NDM-1._ However, this study reports the first identification of *A. baumannii* strains co-harboring these carbapenemase genes in Brazil. The co-occurrence of the carbapenemase genes *bla*_OXA-58_ and *bla*_NDM-1_ in clinical strains of *A. baumannii* is relatively uncommon. To the best of our knowledge, the 12 co-producing strains identified in this study represent the highest number reported in a single investigation. The spread of plasmids harboring critical antimicrobial resistance genes may facilitate the emergence of bacterial strains resistant to several classes of antibiotics, thereby presenting new challenges for the treatment of infectious diseases. Thus, the results presented highlight the propensity of *A. baumannii* to acquire and accumulate resistance genes to significant antimicrobial classes utilized in clinical practice. The characterization of MDR-CRAB strains is crucial for monitoring the evolution of resistance, with the aim of containing their rapid global spread, preventing outbreaks, and optimizing therapy.

## Data availability statement

The datasets presented in this study can be found in online repositories. The names of the repository/repositories and accession number(s) can be found in the article/Supplementary material.

## Author contributions

DR: Conceptualization, Formal analysis, Investigation, Methodology, Visualization, Writing – original draft, Writing – review & editing, Software. MS: Conceptualization, Formal analysis, Investigation, Methodology, Visualization, Writing – review & editing, Software, Writing – original draft. BP: Validation, Writing – review & editing. BK: Methodology, Writing – review & editing. RP: Methodology, Writing – review & editing. GK: Methodology, Writing – review & editing. FP: Methodology, Writing – review & editing. RiL: Methodology, Writing – review & editing. AS: Methodology, Writing – review & editing. RoL: Methodology, Writing – review & editing. EM: Methodology, Writing – review & editing. CR: Conceptualization, Data curation, Investigation, Methodology, Supervision, Validation, Writing – review & editing. AC-A: Conceptualization, Data curation, Formal analysis, Funding acquisition, Investigation, Project administration, Resources, Supervision, Validation, Writing – review & editing.
