# Supplementary material for: Genomic study of Acinetobacter baumannii strains co-harboring blaOXA-58 and blaNDM-1 reveals a large multidrug-resistant plasmid encoding these carbapenemases in Brazil
Source: Front Microbiol. 2024 Jul 17;15:1439373. doi: 10.3389/fmicb.2024.1439373 (PMC11288812; doi:10.3389/fmicb.2024.1439373)
Supplement: Supplementary file 1 [file Data_Sheet_1.PDF]

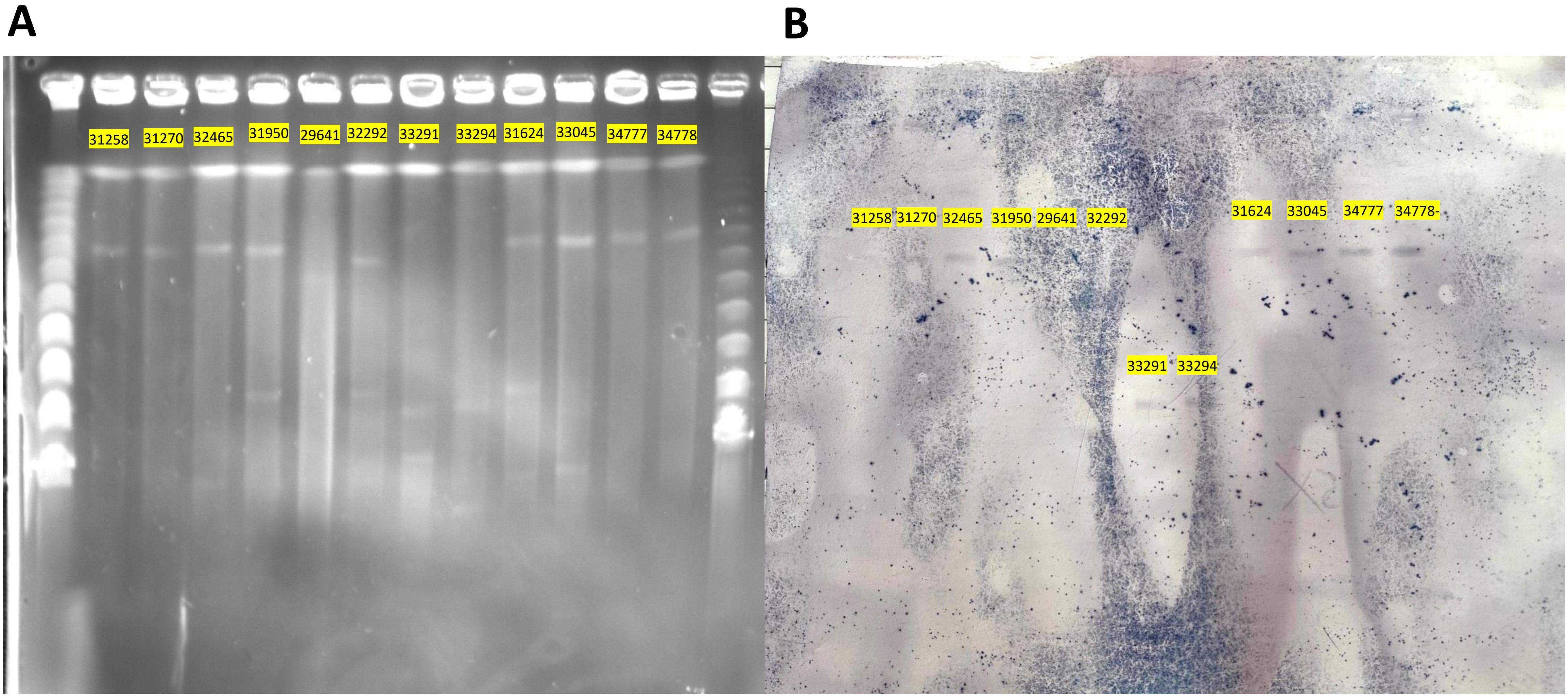

Figure 1: (A) S1-nuclease pulsed field gel electrophoresis; **(B)** Southern blot membrane after hybridization with the *bla*$_{OXA-58}$ probe.

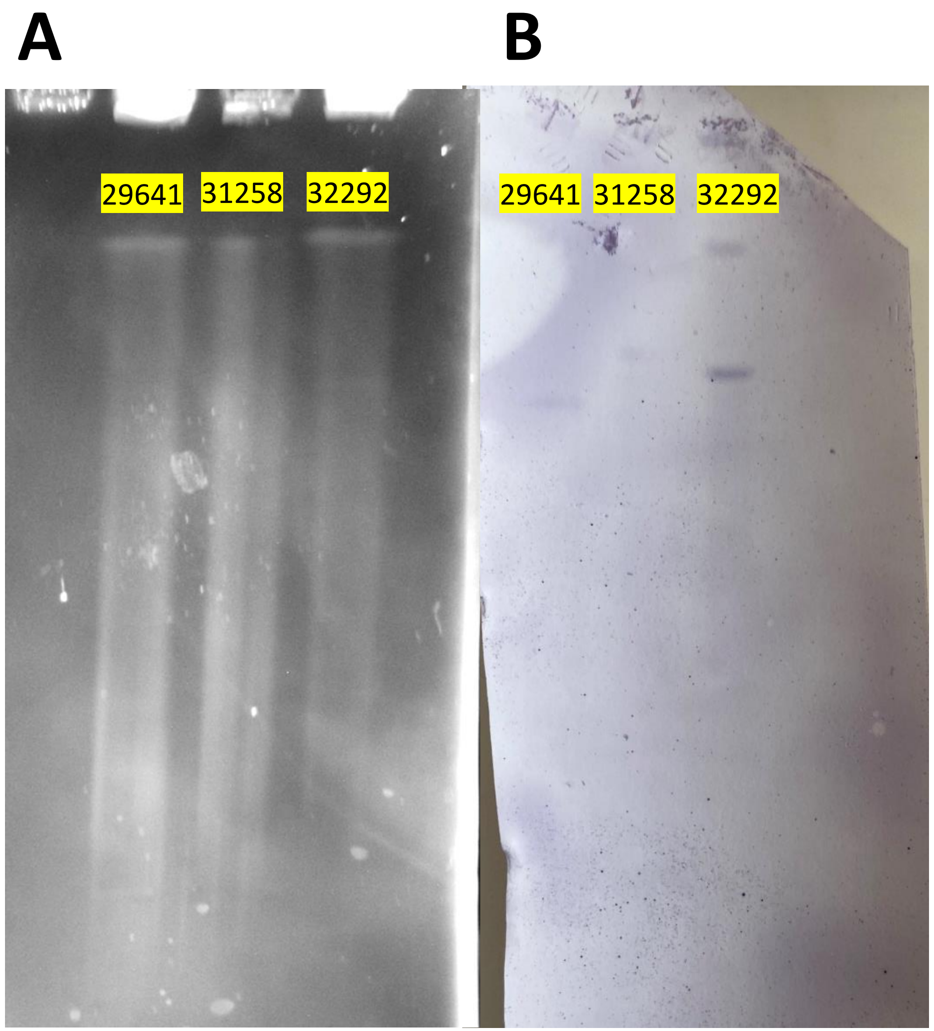

Figure 2: (A) S1-nuclease pulsed field gel electrophoresis; **(B)** Southern blot membrane after hybridization with the *bla*$_{OXA-58}$ probe.

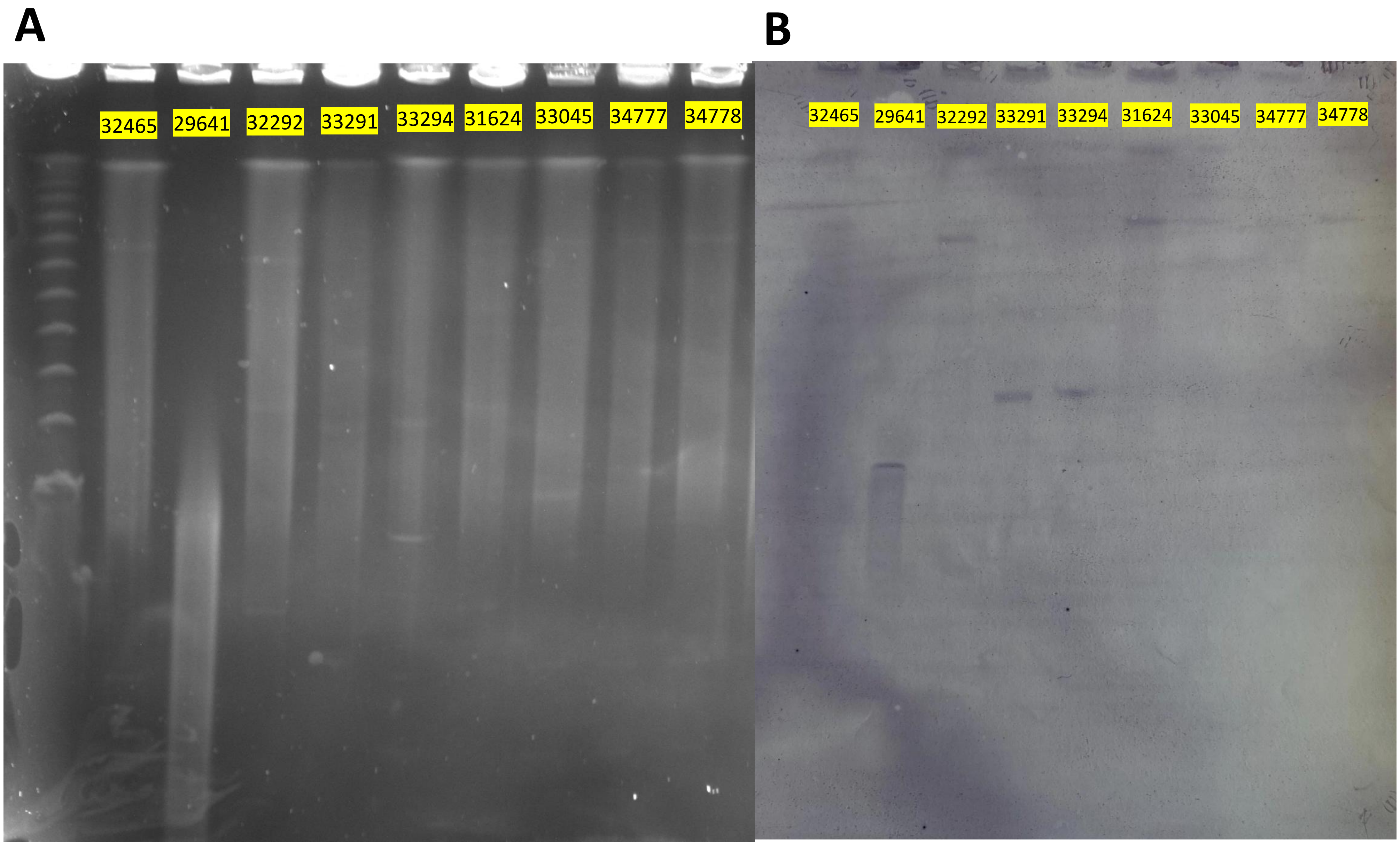

Figure 3: (A) S1-nuclease pulsed field gel electrophoresis; **(B)** Southern blot membrane after hybridization with the *bla*$_{\text{NDM-1}}$ probe.

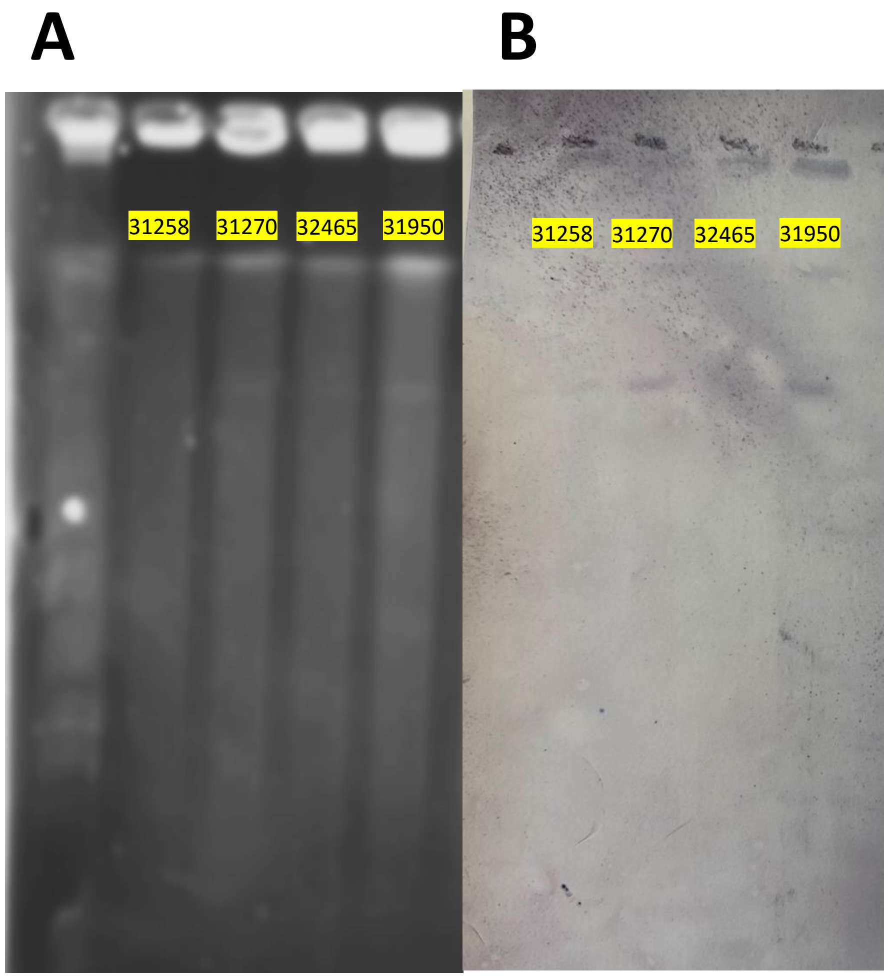

Figure 4: (A) S1-nuclease pulsed field gel electrophoresis **(B)** Southern blot membrane after hybridization with the $\textit{bla}_{\text{NDM-1}}$ probe.
